# The development of a global Midwifery Education Accreditation Programme

**DOI:** 10.1080/16549716.2018.1489604

**Published:** 2018-07-04

**Authors:** Andrea Nove, Sally Pairman, Leah F. Bohle, Shantanu Garg, Nester T. Moyo, Michaela Michel-Schuldt, Axel Hoffmann, Gonçalo Castro

**Affiliations:** a Novametrics Ltd, Duffield, Derbyshire, UK; b International Confederation of Midwives, The Hague, Netherlands; c Swiss Centre for International Health, Swiss Tropical and Public Health Institute, Basel Switzerland and University of Basel, Basel, Switzerland; d Centre for Midwifery, Child and Family Health, Faculty of Health, University of Technology Sydney, Sydney, Australia; e Department of Education and Training, Swiss Tropical and Public Health Institute, Basel Switzerland and University of Basel, Basel, Switzerland

**Keywords:** Midwifery regulation, quality of care, higher education, maternal and newborn health, accountability

## Abstract

**Background**: Many countries are responding to the global shortage of midwives by increasing the student intake to their midwifery schools. At the same time, attention must be paid to the quality of education being provided, so that quality of midwifery care can be assured. Methods of assuring quality of education include accreditation schemes, but capacity to implement such schemes is weak in many countries.

**Objective**: This paper describes the process of developing and pilot testing the International Confederation of Midwives’ Midwifery Education Accreditation Programme (ICM MEAP), based on global standards for midwifery education, and discusses the potential contribution it can make to building capacity and improving quality of care for mothers and their newborns.

**Methods**: A review of relevant global, regional and national standards and tools informed the development of a set of assessment criteria (which was validated during an international consultation exercise) and a process for applying these criteria to midwifery schools. The process was pilot tested in two countries: Comoros and Trinidad and Tobago.

**Results**: The assessment criteria and accreditation process were found to be appropriate in both country contexts, but both were refined after the pilot to make them more user-friendly.

**Conclusion**: The ICM MEAP has the potential to contribute to improving health outcomes for women and newborns by building institutional capacity for the provision of high-quality midwifery education and thus improved quality of midwifery care, via improved accountability for the quality of midwifery education.

## Background

The global shortage of health workers in general, and of midwives in particular, is well acknowledged, as is the fact that investment in the health workforce is key to the achievement of the Sustainable Development Goals (SDGs) [1,2]. Many countries have responded to the shortage of midwives by increasing the number of midwife education programmes and available student places [2]. However, increasing the size of the midwifery workforce only addresses the availability of midwives. It is also necessary to take into account the quality of care that they are competent and enabled to provide, otherwise increased availability may not result in improved health outcomes [3,4].

Quality of care is a multi-dimensional concept, which demands a fit-for-purpose workforce operating within an enabling environment [1,5]. One of the foundations of quality of care is the provision of pre-service education that ensures entry-level health workers have the essential competencies to meet the needs of the population they serve. However, the quality of midwifery education varies both within and between countries [6,7], and poor quality of education has been identified as a barrier to high-quality midwifery care [8].

It is the responsibility of regulatory organisations to ensure that health professionals develop and maintain the necessary competencies to provide high-quality health care, and this includes the accreditation of education programmes, which is acknowledged as an important strategy for improving the quality of midwifery care [9,10]. Accreditation is formal recognition that an education institution meets prescribed standards to ensure that its graduates achieve a minimum level of competency [11]. In most countries, national regulatory mechanisms exist to license and/or register midwives [2], but education accreditation mechanisms do not exist in all countries, and where they do exist they are often weak [6,11]. This is because regulators often lack the resources, authority and technical capacity to be fully effective, and/or the education standards to which they work are out-of-date and do not align with global standards [6,12].

In 2013, the World Health Organisation (WHO) recommended that there should be international efforts to ensure that health worker education in all countries is properly regulated and guided by global standards [12]. Such efforts have been made for medical education: the World Federation for Medical Education first published a guideline for basic medical education in 2005, which was designed to be used as a basis for accreditation of pre-service education programmes [13]. In 2016, there was a call for international efforts to improve the regulation of midwifery education [14]. Although global standards exist for midwifery education [15], a standard global accreditation system has not been designed around them (although some regional and national standards do exist [16–18]). Conscious of this and of the pressing need for investments in quality midwifery care – especially in low- and middle-income countries – in order to achieve the health-related SDGs [2], the International Confederation of Midwives (ICM) has developed a global Midwifery Education Accreditation Programme (MEAP) in collaboration with the Swiss Tropical and Public Health Institute (Swiss TPH).

In 2017, the ICM MEAP was designed and pilot tested in two countries: Comoros and Trinidad & Tobago. The aim of this paper is to describe the process of developing the ICM MEAP, how it works, and the experience of applying it in the two pilot countries, and in the light of this experience, to consider its potential for helping to build capacity for high-quality midwifery education in low- and middle-income countries.

## Developing the ICM MEAP

In the design of an initiative such as the ICM MEAP, there is the potential for tension between the desire for global harmonisation and the need for adaptability to the local context. The ICM MEAP has been developed with a philosophy of ‘global principles with context specificity’ [11], i.e. balancing the need for a basic level of consistency with the flexibility to adapt midwifery education to national and sub-national contexts. The decision about whether or not to apply for accreditation under the ICM MEAP is the school’s – ICM MEAP accreditation is a voluntary process.

The ICM MEAP is designed to be applicable to pre-service midwifery education programmes whether they are direct entry or post-nursing, because ICM global standards for midwifery education apply to both direct-entry and post-nursing midwifery programmes. The MEAP does not, however, apply to other types of midwifery education programme such as apprenticeships, postgraduate programmes or dual nursing and midwifery programmes. ICM global standards for education set a benchmark for programmes that produce midwives able to meet all the ICM competencies and provide high-quality midwifery care. There is a wide variety of midwifery programmes across the world with differences in theory and practice hours, alignment to the ICM competencies and qualification types. Combined nurse midwifery programmes that aim to produce both nurses and midwives from a single combined programme of 2–4 years’ duration are not eligible for the MEAP. Many countries that use them are considering how to separate the professional education of nurses and midwives so that both can be strengthened. The ICM standards, and by extension the MEAP, encourage such changes.

**Figure 1.**
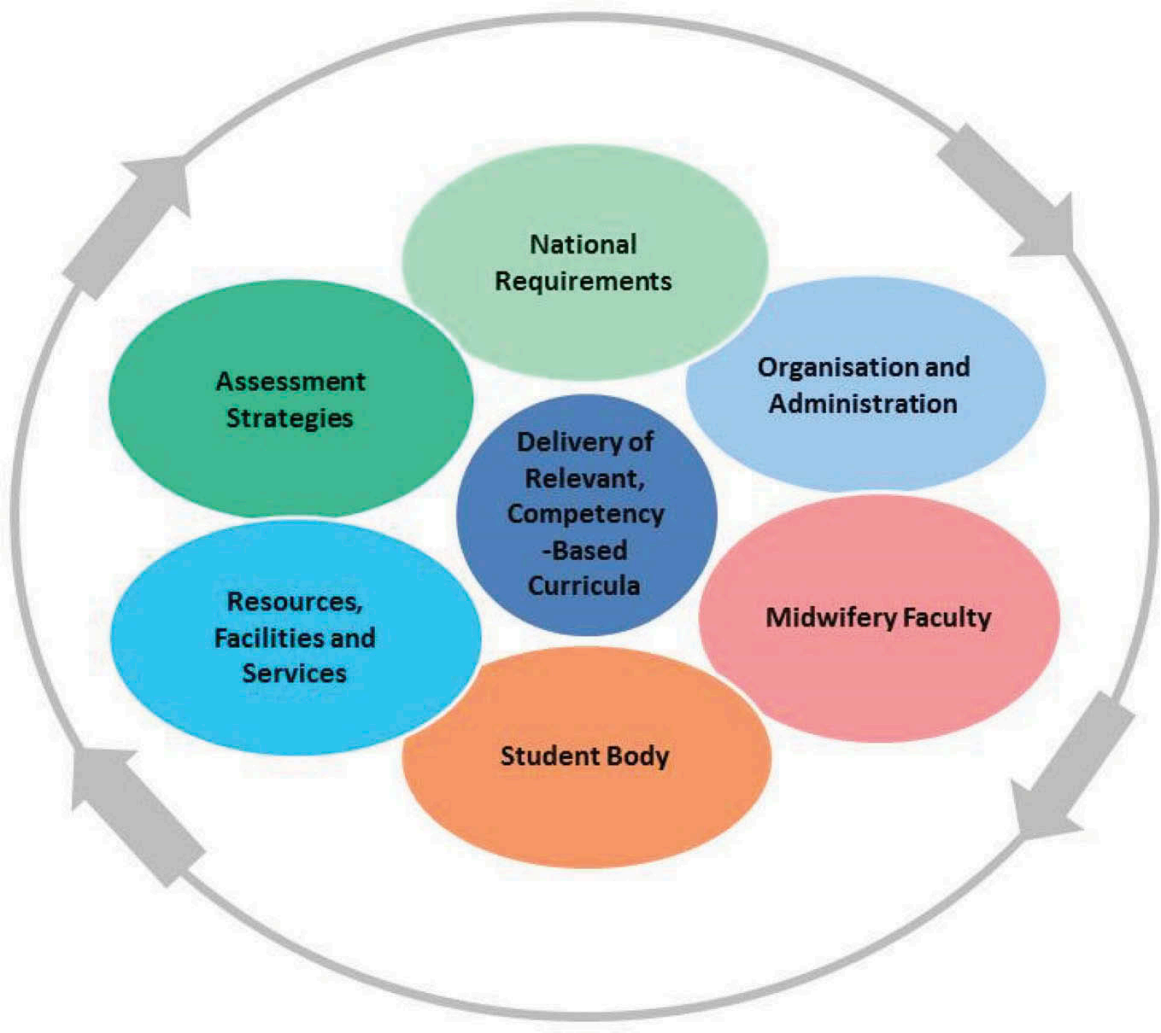
Seven categories of assessment criteria. Source: ICM MEAP, adapted from IntraHealth School Management Improvement Framework [29].

The process of developing the ICM MEAP was guided by evidence and experience from midwifery and other professions, and occurred in 5 stages:

1. *The development of a set of assessment criteria, informed by a desk review of global, regional and national guidance and standards relevant to midwifery education*. A search was conducted using the keywords ‘midwi*’ and ‘education’ and ‘regulation’ and/or ‘accreditation’ using the Google Scholar and PubMed databases. Searches also included websites such as those of WHO (global and regional sites), United Nations Population Fund (UNFPA) and ICM. Selected midwifery regulatory bodies around the world were contacted to request relevant documents relating to midwifery education accreditation. The documents located and screened via this process included publications by ICM [15,19–23], WHO [12,24–27], and others [28–31]. Information on assessment structure and criteria derived from the desk review was triangulated and developed into a first draft set of accreditation criteria. These were presented at an online consultative forum involving 12 international midwifery education and accreditation experts from different regions of the world and including representatives of UNFPA and WHO. Feedback from participants was reviewed by two members of the MEAP development team, and incorporated if both reviewers agreed. From this process, 39 main themes emerged, which were organised into seven categories (Figure 1). The structure of the categories follows the structure of ICM’s global standards for midwifery education [15]. For each standard, a number of assessment criteria guide the assessment: there are 183 criteria in total. These assessment questions are classed as ‘basic’, ‘additional’, or ‘criteria of excellence’. The great majority of basic assessment criteria are based on the ICM Global Standards for Midwifery Education and therefore essential for accreditation.
2. *The design of the accreditation process based on the assessment criteria and reference to existing accreditation mechanisms for the education of health professionals*. This stage was informed by a consultation exercise during the 2017 ICM Congress, where progress on the development of the MEAP was presented. After the presentation of the accreditation process, approximately 70 delegates took part in a consultative forum. They were grouped according to their professional backgrounds and preferred language: there were four English-speaking groups: educators, students, regulators and practitioners, and one French-speaking group with participants from a variety of professional backgrounds. During the discussions, which were moderated by Swiss TPH and ICM, participants were asked to describe the potential benefits and challenges of the ICM MEAP, and to present their conclusions back to the entire forum. The content of their presentations was documented. Participants saw the MEAP as an opportunity to identify gaps in education quality, which would allow them to mobilise resources for improving the quality of midwifery education (from domestic funds and/or donors). The potential for international harmonisation of quality standards was acknowledged as an opportunity for improved collaboration between countries (e.g. student and staff exchanges) and increased mobility for these key health workers who are in such short supply in many countries, although the need was also identified for ethical practices in relation to international migration.
3. The *development of an online platform to support the ICM MEAP process*. Based on an analysis of the platform requirements (including business processes modelling) and a market review, Moodle was chosen as the system for the MEAP platform. The system allows institutions applying for accreditation, ICM and those working on the accreditation assessments to exchange information and documentation, as well as archiving of documents.
4. *Training of an international panel of midwifery experts to carry out the accreditation assessments*. Currently, this consists of 8 people, who were nominated by ICM. The training took place over a two-day period at the ICM offices in the Netherlands, followed by participation in the pilot test (see below) for further practical experience. Each expert was given access to the MEAP Criteria Assessment Handbook, which sets out the assessment criteria and includes guidelines for accreditation experts, reference links to key documents, recommended assessment methods, as well as a list of documents recommended for review.
5. *A pilot test in two countries*. In each country, a 4-day visit took place, including briefing and de-briefing meetings in addition to assessment visits. More information about the pilot test is provided later in this paper.

## The ICM MEAP process

**Figure 2.**
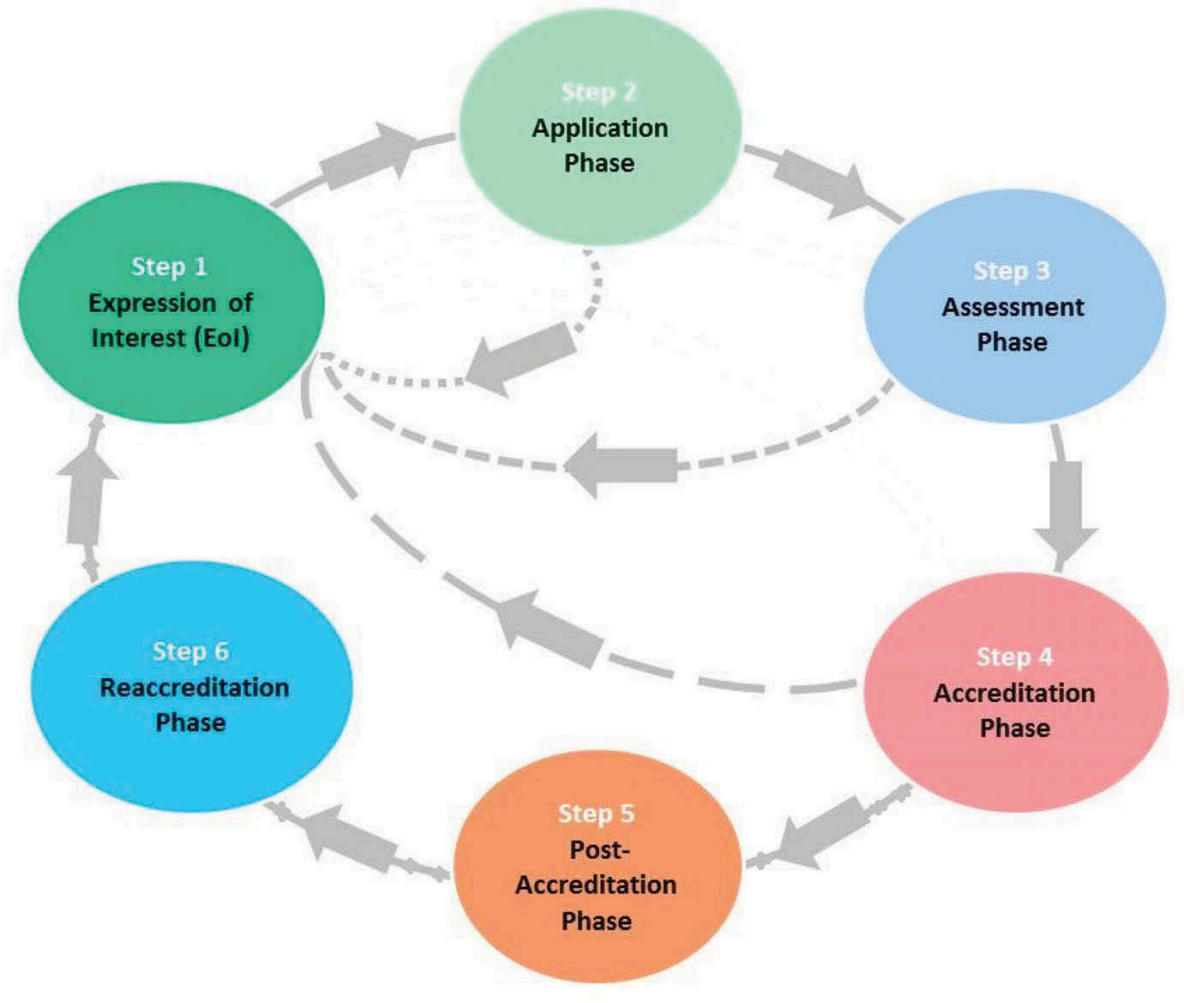
The ICM MEAP process.

Figure 2 illustrates the ICM MEAP process, which is described in more detail below.

### Phase 1: expression of interest

As a first step in the process, an interested educational institution contacts ICM to register their interest and, if needed, discuss the MEAP with an ICM representative. If they wish to pursue the MEAP, institutions are granted access to the ‘expression of interest’ section of the online platform and invited to submit a completed expression of interest form. The form requests various pieces of information, of which the most important are: (1) contact details, (2) whether the institution offers an eligible programme, i.e. pre-service/pre-registration midwifery education pathways that are either three-year direct entry or 18-month post-nursing registration, and (3) whether or not an accreditation/regulatory body exists in the country. The completed form is reviewed by the ICM MEAP coordinator, and if a minimum set of criteria is fulfilled, the institution is invited to move to the next step in the process. ICM sets fees separately for the desk review of the submitted self-evaluation materials and for the site visit and accreditation phase, so an institution only pays the latter if its application progresses to that phase.

### Phase 2: application

The educational institution then submits a detailed self-evaluation form via the online platform, including information about technical competence and management capacity according to the ICM MEAP accreditation criteria. Each application relates to a single education programme, even if the institution offers more than one programme.

### Phase 3: assessment

Two accreditation experts, supported by the ICM MEAP coordinator, examine the completed form and supporting documents. If these indicate that the institution has the potential to be accredited, an in-person site visit is arranged. The site visit starts with a briefing meeting convened by the applying institutions and in collaboration with the ICM accreditation experts. During the site visit, the accreditation experts conduct additional document reviews and hold interviews and focus group discussions with key stakeholders including: representatives of relevant government ministries, senior and junior midwifery faculty, school administrative and library staff, practising midwives, students, graduates and users of midwifery services where appropriate. They also observe teaching and clinical practice sessions and the standard of infrastructure such as classrooms and skills labs.

At the end of the site visit, the experts prepare a draft evaluation report using a structured template, which is presented at one or more debriefing meetings attended by representatives of the educational institution and any stakeholders that the institution wishes to invite. Feedback from the meeting(s) is incorporated into the draft report, which is then circulated for review and further feedback before being finalised. The final report is divided into the main themes and has a particular focus on strengths, aspects to be improved and recommendations. Each summary of main themes is followed by a judgement of both accreditation experts, advising if the programme meets the standards, would meet the standards with minimal further action (requirements) or does not meet the standards. Recommendations can be made for improvements, even if standards are met.

### Phase 4: accreditation

On the basis of the evaluation report, one of three recommendations is made to the ICM Board:

- *Accreditation granted*. Institutions that meet all the basic criteria gain full accreditation that is valid for five years, giving them a certificate, the right to show an ICM accreditation logo on their publications, and inclusion on a publicly available list of ICM-accredited institutions.
- *Accreditation granted with conditions*. Institutions that meet nearly all the basic criteria but need minor improvements to meet basic criteria are granted conditional accreditation, under which the required improvements must be made within set time frames. Once the required improvements are made, and written evidence is submitted and accepted, the programme is accredited for a period of three years, at the end of which another assessment will take place.
- *Accreditation not granted*.

### Phase 5: post-accreditation

The assessment criteria include a continuous quality improvement process, which is designed to ‘build in’ sustainability by ensuring that the school takes responsibility for maintaining the high standards required for initial accreditation. One year before the accreditation period ends, accredited institutions are invited by ICM to apply for re-accreditation.

### Phase 6: reaccreditation

Programmes that apply for re-accreditation will repeat the main accreditation process (assessment phase – self-evaluation and site visit). If they can demonstrate that the standards that gained them accreditation in the first place have been maintained, they are re-accredited for a further five years. ICM is considering a desk-review process for any subsequent re-accreditations when programmes have continued to meet the standards.

## Pilot testing the ICM MEAP in Comoros and Trinidad & Tobago

After receiving information about the MEAP from ICM, midwifery schools in Comoros and Trinidad & Tobago volunteered to act as pilot sites. In Trinidad & Tobago, the pilot took place in September 2017 at the San Fernando School of Midwifery. In Comoros, it took place in October 2017 at l’École de Médecine et de Santé Publique (EMSP) in Moroni.

Both pilot sites were provided with a detailed evaluation report and the opportunity to discuss the content of the report with the ICM accreditation team. The report summarised the recent history of midwifery education in the country and at the pilot site, highlighted the specific strengths of the school and the challenges currently being faced in relation to the seven categories of assessment criteria (Figure 1), and made specific recommendations about how to address the identified challenges.

The pilot showed that the overall structure and content of the tools and documentation is sound, but in certain specific places there was a need for clarification of the wording used (e.g. some of the questions on the application form were misunderstood), and these improvements have now been made and the documentation finalised. In addition, a number of lessons were learned about applying the process, as follows:

- The handbook issued to the ICM accreditation experts required restructuring to make it more user-friendly and easier to follow during the site visit, and the data collection tools used during the site visit needed more space to describe overall findings for each theme and sub-theme in the handbook. This work has been completed, and revised versions of these documents are now available. Although the original documentation was available in both English and French to reflect the languages used in the pilot sites, the updated documentation is currently available only in English. Funding is being sought to provide translation into French, Spanish and possibly other languages.
- The pilot process highlighted the difficulty in using a quantitative scoring scheme to assess programmes against the criteria, which led to the decision not to apply such a scoring scheme to the assessment. Because each programme was contextually different, the professional judgement of the accreditation experts was important in weighting the importance of various factors and qualitative methods were judged to more appropriate than quantitative. Whilst it was possible to make a baseline assessment of whether or not a programme met each standard and thus whether or not accreditation should be granted, it was not possible to use a scoring system to identify standards of ‘above average’ or ‘excellence’. Examples of excellence could be identified for some criteria, but these examples were not necessarily reflected across the whole programme.
- Additional time should be set aside for the assessment team to understand better the local/country context; this was realised by including a ‘country context’ section in the MEAP Assessment Criteria Handbook and evaluation report to gather important information, and by building in an early meeting with government/regulatory stakeholders who are in a position to provide detail on the wider, systemic issues that may influence the performance of the school.

The pilot also confirmed some hypotheses, as follows:

- Some of the documentation that is required to accompany the application for accreditation is difficult to transmit electronically due to large file sizes and/or poor Internet connections, which means it cannot always be reviewed by the assessment team in advance of the site visit. In such cases, additional time should be set aside during the site visit for the assessment team to review important documentation that was not possible to transmit in advance.
- The assessment team should consist of two or three accreditation experts. This is enough to make an objective assessment but not so many that the visit becomes disruptive to the running of the school. Because it was a pilot, there were more people than this in the assessment teams, and whilst this was necessary to ensure a thorough pilot test, it was sometimes disruptive to clinical areas and a logistical burden for the institutions.

The pilot was designed to cover phases 1 to 3 of the process, i.e. up to and including the assessment phase. It did not include the accreditation, post-accreditation or reaccreditation phases because it aimed to test the processes and documents rather than to assess whether or not the pilot sites met the accreditation criteria. Furthermore, ICM MEAP documentation suggests allowing 3–6 months for a school to prepare all the documentation and make arrangements for a site visit, whereas the pilot sites had only a few weeks. To build on this early piloting work, later in 2018 ICM intends to call for interest from midwifery schools with eligible programmes (that are likely to meet the standards) to undertake the MEAP. A thorough evaluation is planned to be undertaken alongside these next applications of the MEAP, and this evaluation may lead to amendments to the MEAP before full roll out.

## The Midwifery Education Development Pathway (MEDPath) (working title)

The pilots confirmed that pre-service midwifery education programmes do not always meet the ICM global standards and that there is not currently a process to support educators and educational institutions to make the changes that would be required for accreditation to be achieved. It is likely that educators will recognise this outcome during the self-evaluation process and if not, that the accreditation experts will reach this conclusion during the desk review and will advise accordingly. Schools may ‘opt out’ as soon as it becomes clear that they do not meet the standards, in which case they are not required to bear the costs of a site visit.

When this happens, ICM wants to provide an alternative pathway for schools to follow. ICM is planning to develop a series of online resources and a structured process of support, called the Midwifery Education Development Pathway (MEDPath), to assist midwifery programmes that do not meet the global standards for midwifery education to make the changes necessary to improve the standard and quality of the programme. The ICM MEDPath will be a separate process to the ICM MEAP but its future development will provide practical support and guidance to midwifery educators seeking to remediate midwifery programmes and improve standards and quality. ICM is currently seeking funding to begin development of the MEDPath.

## Discussion

The ICM MEAP has been developed by international experts in midwifery education and accreditation, and is grounded in up-to-date evidence. It therefore represents the latest thinking on excellence in midwifery education, and has been pilot tested in two countries. Assessment of institutional performance against ICM MEAP criteria means that midwifery education providers and stakeholders (e.g. Ministry of Health) can be very clear about their strengths and weaknesses, and what they need to do in order to provide the highest-quality midwifery education.

If its application can be scaled up, the ICM MEAP has the potential to make a significant contribution to building capacity for high-quality midwifery education. Site visits give schools the opportunity to discuss issues specific to their country/region/school with international experts, who can share their experience and expertise to help build capacity within the country, and also learn themselves about how midwifery education works in countries/contexts other than their own.

A system for accreditation of education programmes can be thought of as a type of accountability mechanism that aims to improve the performance of education institutions and their programmes (and thus the quality of care provided by their graduates) by increasing transparency about their technical and managerial capacity [32,33]. A good accountability mechanism fosters an environment of learning and improvement (as opposed to an environment of blaming and shaming) [34], and this is the central philosophy of the ICM MEAP.

In many countries, health professional regulatory bodies are responsible for ensuring that there is a functional system for the accreditation of education institutions. Although the ICM MEAP is entirely voluntary, in some countries it is possible that regulators may consider adopting it as a requirement. Conversely, there may be professional resistance to the ICM MEAP in countries that already have a national accreditation process that requires strengthening: current accreditation bodies may view it as a threat. Involving them in the assessment and site visit is a possible solution and capacity building opportunity. Experience in Afghanistan has demonstrated that this is feasible even in low-resource settings [35].

Currently, evidence indicates that private medical schools are less likely to undergo accreditation procedures than publicly funded schools [11], and it seems reasonable to speculate that the same would be true of midwifery schools. Linking registration to graduation from an accredited education provider would provide an incentive for schools to seek accreditation. In countries with a large private education sector, this could be a way to ensure that both public and private sectors are committed to providing high-quality education (and thus being accountable to their students and to the women and children who will be served by them in the future), and to encourage greater harmonisation and collaboration between the public and private sectors.

As well as greater collaboration between sectors, rolling out the ICM MEAP more widely will create opportunities for collaboration and cooperation between schools and countries, as those who have already been accredited can help to build capacity among those yet to do so. Additionally, the inclusion of criteria of excellence in the MEAP framework provides the opportunity to collate information about best practice, and for excellent schools to showcase their strengths and inspire change both within and beyond their borders. For example, schools that offer high-quality specialised midwifery education could publicise this fact via their accredited status. There is a growing trend for the establishment of Centres of Excellence in higher education [36,37], and the ICM MEAP provides the opportunity to link to these related initiatives.

The 2014 Lancet Series on Midwifery provided evidence that scaling up midwifery will make a massive contribution to ending preventable maternal and newborn mortality [38]. It is, however, important to note that these estimates were made using the Lives Saved Tool (LiST), which works on the assumption that reproductive, maternal and newborn health interventions will be delivered at a specific quality relative to overall coverage levels [39]. Therefore, addressing effective coverage of midwives without addressing quality of care may mean that the impact of scaling up midwifery could be muted. The ICM MEAP provides an opportunity to improve quality of care and thus helps to ensure that the potential of scaling up midwifery is fulfilled.

Recent increases in levels of health worker migration [40] have led to calls for international oversight of key elements of health worker regulation such as education accreditation [41], so that destination countries can be sure that in-migrating health workers have the required competencies, health workers have more choices about where they can work, and it becomes easier to deploy key health workers to where they are most needed. The ICM MEAP provides an opportunity to harmonise education standards. Of course, countries with a history of ‘brain drain’ may be reluctant to make it easier for their health professionals to emigrate, so work to address known ‘push’ factors is needed alongside work to improve the quality and harmonisation of pre-service education, and it will be important for destination countries to adhere to ethical international recruitment policies [42]. It is also worth noting that patterns of migration are changing with increased blurring of ‘source’ and ‘destination’ countries [43].

The ICM MEAP aligns well with the Unified Accountability Framework (UAF) for the Global Strategy for Women’s, Children’s and Adolescents’ Health [44] and its three stages of monitor, review and remedial action. A strong accountability mechanism includes a recourse mechanism, i.e. a process that can be triggered if the entity being held to account does not take the necessary remedial action identified during the monitoring and review phases [34]. In the case of the ICM MEAP, this recourse mechanism is the withholding of accreditation or reaccreditation. Furthermore, the process can be used by the education institutions to hold government and other stakeholders to account, e.g. if the assessment concludes that there are specific gaps in provision, the institution can use this evidence to advocate for more resources to be targeted at the identified gaps. This alignment with global accountability architecture means that the ICM MEAP has the potential to make a significant contribution to improving accountability for the health of women and newborns.

## Conclusion

The ICM MEAP aims to bring about increased capacity for the provision of high-quality midwifery education and thus improved quality of midwifery care, via improved accountability for the quality of midwifery education. A pilot test in two countries has demonstrated that the process is appropriate for midwifery education institutions in different contexts. Funding is now being sought by ICM to scale up the implementation of the MEAP, and as part of this process there are plans to carry out a robust evaluation of its impact on the quality of midwifery education and the quality of midwifery care. The policy context is a crucial consideration: a nationally owned accreditation system can only be established if there is an appropriate policy foundation [35]. Education accreditation is a vital element of effective midwifery regulation, but it is only one element: quality of care will improve if all elements are given attention.

## Paper context

Global standards exist for midwifery education, but there was no global midwifery education accreditation programme (MEAP) until one was developed in 2017 by ICM. This paper describes the development of the ICM MEAP, how it works, and the experience of piloting it. Midwifery schools, ministries of health/education, and regulatory bodies in low- and middle-income countries are encouraged to consider using the MEAP process to build capacity and improve accountability for high-quality midwifery education.
